# Using Outcome Mapping to Mobilize Critical Stakeholders for a Gender Responsive Rift Valley Fever and Newcastle Disease Vaccine Value Chain in Rwanda

**DOI:** 10.3389/fgwh.2022.732292

**Published:** 2022-04-19

**Authors:** Tess Gannaway, Denis Majyambere, Mary Kabarungi, Liberata Mukamana, Fidèle Niyitanga, Janna Schurer, Beth Miller, Hellen Amuguni

**Affiliations:** ^1^Department of Infectious Disease and Global Health, Cummings School of Veterinary Medicine at Tufts University, North Grafton, MA, United States; ^2^College of Agriculture, Animal Sciences and Veterinary Medicine, University of Rwanda, Nyagatare, Rwanda; ^3^College of Business and Economics, University of Rwanda, Kigali, Rwanda; ^4^Center for One Health, University of Global Health Equity, Kigali, Rwanda; ^5^Miller Consulting, Inc., Little Rock, AR, United States

**Keywords:** livestock vaccine accessibility, gender, stakeholders, women, smallholder farmers

## Abstract

Approximately 752 million of the world's poor keep livestock to produce food, generate income, and build assets. Women represent two-thirds (~400 million people) of low-income livestock keepers. Infectious diseases are a major issue in preventing livestock keepers from optimizing production earnings and improving food security. In Rwanda, highly contagious yet preventable diseases that affect animals that women manage, such as Rift Valley fever in goats and Newcastle disease in chickens have a high-mortality rate and can devastate their herds. Women are disproportionately affected because they bear primary responsibility for goats and chickens. These diseases are preventable through vaccination, but smallholder women farmers rarely benefit from livestock vaccines. Social norms and entrenched cultural stereotypes limit women's confidence and decision-making and restrict their access to resources and information. Women smallholder farmers find that there is little support for the small livestock they manage, because of the official preference given to cattle. They are also challenged by limited availability of livestock vaccines due to lack of a cold chain, inadequate extension, and veterinary services, especially for goats and chickens, and unreliable structures for vaccine delivery. To identify opportunities for women's engagement in the livestock vaccine value chain (LVVC) and reduce their barriers to accessing and using livestock vaccines, we used Outcome Mapping, a stakeholder engagement tool, and the Gender Equality Continuum Tool to classify and engage critical partners in the LVVC. We analyzed each critical partner's capacities, incentives, and drivers for engagement with women, challenges and barriers that hinder their support for women farmers, opportunities at systemic and programmatic levels for women's participation and benefit in the LVVC, and the gender capacities and perceptions of different stakeholders. Enhanced positioning and visibility of women in the LVVC can occur through a systemic engagement of all stakeholders, and recognition of the roles that women play. Women smallholder farmer involvement when determining and shaping the potential entry-points is critical to ensure support for their existing responsibilities in family food security, and future opportunities for generating income. Strengthening gender capacities of LVVC stakeholders, addressing identified barriers, and building on existing opportunities can increase women's participation in the LVVC.

## Introduction

Worldwide, ~752 million people rely on livestock as their main source of income, sustenance and liquid capital. The depth of poverty among livestock keepers is particularly high in sub-Saharan Africa, where it is estimated that more than 85 percent of poor livestock keepers live in extreme poverty (1). The majority (two-thirds) are women (2), who rely on their animals more heavily than their male counterparts for sustenance (3). Female livestock keepers tend to own more small ruminants (goats, sheep, etc.) and poultry than large livestock (water buffalo, cows, etc.) (4, 5). A lack of legal access to land for large ruminant rearing and grazing, the ease of physically restraining smaller animals, opportunities for informal ownership, and minimal required inputs lead women in developing countries to prioritize these species (6).Women have limited access to services, credit, technology, training, and information regarding livestock, putting them at greater risk of animal death or low productivity (7). They have less access to markets and cooperatives and income generated from livestock compared to men (8). Unpaid care work, food preparation, home sanitation, and acquiring fuel and water (9, 10) limits the time available for women to manage and market their livestock, further increasing their risk of losing small ruminants and poultry to infectious disease. However, much of the animal-associated disease burden is preventable through vaccination.

Empowering female livestock keepers and decreasing the number of animals lost to infectious disease are development goals that show great promise for improving the lives of livestock and women (11). Families of such women also benefit as women dedicate ~90% of their income from agricultural production to their families, while men spend only 30–40% (11). Empowering female farmers, especially rural subsistence farmers, has been shown to be an effective means of fighting household hunger and poverty (12) and gender equality remains a top developmental priority in the 2015 United Nations Sustainable Development Goals (11). The growing demand for protein globally (12) along with the gendered effects of climate change further illustrate the need to empower women within the livestock sector (11). Ensuring women's access to livestock vaccines and participation within livestock vaccine production and distribution in Rwanda is a key intervention to achieve this goal. Reducing gender barriers to women's active participation along the Newcastle disease (ND) and Rift Valley fever (RVF) vaccine distribution and delivery chain as service providers, distributors and users will empower women to benefit from livestock vaccines and improve livestock productivity.

In Rwanda, as in many African countries, 75–90% of small-scale poultry farmers are women, and their flocks are frequently decimated by ND (1). Additionally, goats often die due to (RVF), despite the existence of highly effective vaccines. Obstacles to vaccine uptake and use among small scale farmers include lack of knowledge, low accessibility in rural areas, and a delivery system that prioritizes large scale production and cattle over small-scale poultry and goat production. Women have more difficulty accessing the money to buy vaccines if they want them and have a difficult time traveling to towns where vaccines are sold because of time intensive domestic chores and cultural attitudes restricting women's mobility and decision-making (13).

The Government of Rwanda (GoR) and its supporting institutions are responsible for the development of strategies and legislative framework that guide the manufacture and registration of vaccines including access of these vaccines to small-scale farmers (14). The state is therefore accountable for the implementation of the relevant control measures. RVF vaccine distribution is controlled and regulated by the government (14). ND vaccines are available through the private sector, but are used almost exclusively by large scale commercial poultry producers. Smallholder rural farmers do not have a network for vaccine delivery. The tensions lie in diseases that are important at the farm or village level, but not prioritized for state intervention. For instance, RVF is notifiable, meaning it must be reported to the state (14), but all control measures including vaccination are the responsibility of the owner(s). Government regulations play a key role in vaccine delivery to all end users, but current public funding and policies support large, commercialized livestock production, with minimal recognition of the importance of women's small-scale poultry and goat production for improving family welfare and women's own self-respect and empowerment (12).

Women's role and contribution in the livestock industry must become more visible and prioritized to gain adequate support for improvement. Rwanda's policy environment provides a good foundation. In the World Economic Forum's most recent Global Gender Gap Report, Rwanda is ranked sixth in gender equality amongst all the countries in the world (15). Women hold 61% of the seats in the lower house of Rwanda's national legislature, the largest percentage of any country, while in the United States, it is only 23% (16). The Rwandan constitution includes supportive foundational language and policy to promote gender equality (12). Despite the country's progressive policies and laudable female representation within government, female farmers continue to face the same challenges and marginalization experienced by women around the world. In Rwanda, the agriculture sector contributes 31% of the country's GDP and employs 80% of the female labor force, but women continue to experience less support than men within the sector (12). In the most recent Gender and Youth Mainstreaming Strategy report published by the Rwandan Ministry of Agriculture and Animal Resources, the government identified lack of gender disaggregated data, as well as low capacity to implement gender sensitive policies as major challenges to women's empowerment (12). A low level of financial inclusion, low participation in the most lucrative parts of the agriculture sector, limited access to extension support, inputs, and technologies (including livestock vaccines), and limited control over resources and decision making within the household are also noted as challenges faced by female livestock keepers in Rwanda (12).

A gender-sensitive livestock vaccine value chain (LVVC) analysis is used to identify bottlenecks in the entire system, and specifically places where women's participation is low, allowing strategic interventions for women's inclusion and promotion of gender equality. The supply chain runs from vaccine manufacturing, through distribution, and delivery all the way to the livestock farmer/end user including the policy and regulatory context (17). The LVVC analysis helps identify and enact improvements to the regulatory environment and promote systemic transformation of those gender norms that harm families, communities and nations. A gendered LVVC analysis identifies all stakeholders, systems and processes that would impact men and women smallholders' individual and collective opportunities. Livestock value chain interventions have been used to understand actors in livestock production systems and opportunities for improvement (18), but a gendered analysis has not yet been employed in order to increase vaccine accessibility and adoption by women smallholder farmers, and boost family health benefits and empowerment prospects.

Increasing female livestock keepers' access to livestock vaccines can contribute to women's empowerment, as envisioned by the GoR Constitution and agricultural policy. This Outcome Mapping through stakeholder engagement exercises identified the key actors, their capacities, and incentives to empower women, and additional drivers for women's engagement in the livestock vaccine value chain in Rwanda. The stakeholder analysis had the following 4 aims:

1. Use outcome mapping to identify stakeholders, networks, and incentives along RVF and NCD LVVC in Rwanda.
2. Evaluate the level of gender awareness among key stakeholders using the Gender Equality Continuum tool.
3. Identify barriers and opportunities to women's involvement within the LVVC.
4. Gather data regarding stakeholder's perceived challenges, the long term anticipated behavioral changes they could make, and the support they need to ensure engagement and incorporate women into the LVVC.

## Materials and Methods

The two main tools used to gather information in this study were Outcome Mapping (OM) and the Gender Equality Continuum Tool (GECT).

### Participant Selection

Forty-two participants representing organizations or individuals (17 women and 25 men) acting in the LVVC in Rwanda were invited to participate in an OM stakeholder engagement exercise to identify roles, challenges, incentives, and drivers of key actors for women's engagement and inclusion. The study team initially compiled a list of 32 potential participants from different organizations/institutions. Some organizations were represented by more than one person. The study team then made individual phone calls and office visits to engage stakeholders and invite them to participate. Using a snow balling process, they asked potential participants to recruit other people they considered relevant for this process. The final number of identified potential participants was fifty-six. The list was narrowed down to 42 by eliminating multiple people representing the same organization and playing the same role. Groups representing farmer organizations, women groups, and cooperatives had an average of 4 participants, while other higher level regulatory bodies and private sector institutions had only one participant. Since the tool used- outcome mapping, is very intense and requires focus group like facilitation, <45 participants were considered appropriate to effectively achieve desired outcomes. The participant selection process consisted of two steps: (i) identification of the stakeholders involved in LVVC and their relationships (Who does what? Why? How? And with who?); and (ii) determination of the perceived level of influence of the stakeholders on the LVVC. Participants were chosen purposively to represent stakeholder groups of the LVVC based on their current or anticipated roles in the LVVC and included government, private and non- government sectors at national level. Participants included vaccine regulators, producers, distributors, deliverers, end users and farmers support organizations such as cooperatives, groups with expertise in promoting gender equality, providing financial credit to women, and women support networks. Informed consent was obtained prior to participating in the activities.

Table 1 below provides the different organizations/institutions/individuals who participated, and their roles along the LVVC. Some organizations supported more than one participant.

**Table 1:** Organizations engaged in the Rwanda LVVC stakeholder mapping exercise in November 2019.

**Regulators**	**Importers/distributors**	**Deliverers and end users**	**Support organizations**
Rwanda Agricultural Board and Animal Resources Development Board (RAB)	AGROTECH LTD	Zamura Feeds	Rwanda Poultry Industry Association
Food and Drug Authority (FDA)	Saura-AGROVET LTD	UZIMA Chicken	Heifer International (P)
Rwanda Council of Veterinary Doctors (RCVD)	AVI Farm Solutions	SHEMA	Development Bank of Rwanda
Gender Monitoring Office (GMO)	Mega Vet	Farmer cooperatives	Farmer cooperatives
Ministry of Gender and Family Promotion (MIGEPROF)	Eden Business Center	Technical and Vocational Educational and Training schools (TVET)	TVET schools
Ministry of Agriculture and Animal Resources (MINAGRI)	Green Age International	Kigali Golden Farm	Business Development Fund
National Women's Council	Good Man Pharmaceuticals (D)	Iruganga Imbaraga	SNV Netherlands
	Vet and Corp Limited	Migisha Farm	Pro Femme Twese Hamwe
	Apex Biotech	Abusol Ltd	Adventist Development and Relief Agency (ADRA)
	Heifer International	Public and private sector vets	Kenya Commercial Bank
	Rabasco Limited	College of Agriculture, Animal Sciences and Veterinary Medicine, University of Rwanda (CAVM)	University of Rwanda Center for Gender Studies

*D, Distributor; P, Provider*.

### OM Tool

Outcome Mapping (OM) is a structured participatory tool that uses a bottom-up collaborative process to engage all stakeholders and steer them through an iterative process to recognize their strengths and limitations, their desired change, and how to work collaboratively to bring it about (19). OM has been used successfully across a range of programs, including land use, agriculture, livestock management, infectious disease, health systems, climate change activism, and natural resource management (20). OM creates a framework to guide the integration of interventions into national systems to enhance impact, institutionalization and sustainability and especially supports organizations that have been on the fringe such as women groups to bring their views and strengths to the table. OM provides an opportunity for partnership and collective action to address systemic problems such as poverty, gender inequality, racism, and the global climate crisis. This is because OM gives change makers an applied technique to examine how the world works, and provides a way to organize, connect and move forward in complex contexts, in order to create deep and sustainable societal transformation, between individuals and groups. OM helps change makers to understand and navigate complexity and human behaviors and how they interact with social, economic, and ecological systems. By using the OM approach, change makers become increasingly aware of the vibrant contexts, people, organizations, institutions, and capacities of their interventions (21).

It is a qualitative participatory process that allows different stakeholders to collaborate in a systems analysis. In our study, we used it as a tool to map and track critical changes in the cultural practices, organizational systems, institutional and governance policies, and the progress of stakeholders toward the goal of women's empowerment in the LVVC. It is a bottom-up stakeholder driven approach to planning, monitoring, and evaluating social change initiatives. It uses focus group discussions and maps as the main research method (19). The OM tool can help identify stakeholders and their formal and informal interactions. Through a facilitated process, stakeholders work collaboratively to physically map out their roles and interconnections in the LVVC, support mechanisms, as well as existing systems including analyzing their current limitations and gaps (social, political, and cultural norms), challenges, and barriers that they face (both systemic and programmatic). The stakeholders identify challenges and opportunities for women's participation, engagement, and ability to influence legal and governance structures within the LVVC. Understanding the behavior, interests, inter-relations, and intentions of different stakeholders can be used to assess the influence, resources and effect these stakeholders can have on the viability of the intervention.

Through the OM process, participants analyzed the economic, socio-cultural, familial, legal, political, and psychological networks that shape the current LVVC at the micro, meso and macro levels. They identified the key players, their roles and their impact on vaccine distribution, delivery, and use. They assessed the gender mainstreaming or transformational capacities (skills, knowledge, perceptions, attitudes, behavior) of the actors in the LVVC, while together agreeing on a common vision. The OM process resulted in a platform bringing together different stakeholders that impact each other but rarely come together to intentionally cooperate due to institutional divisions. Organizations, relationships, and gaps that have been invisible in the LVVC were identified, recognized and incorporated into a framework for more meaningful interventions based on their different strengths and expertise.

### GECT

The Gender Equality Continuum Tool (GECT) is used to assess gender sensitivity and responsiveness of different stakeholders in the LVVC. The tool, designed originally for the USAID ASSIST project, helps individuals or groups categorize their gender awareness in behavior and policies and then create a path to achieving transformation of gender relations. The GECT provides a snapshot of the position of each stakeholder, to facilitate discussion moving stakeholders toward gender transformative status, which addresses power inequities between women, men, girls, boys, and non- binary gender groups at the highest levels. This allows for (a) a critical examination of inequalities and gender roles, (b) support and create an enabling environment for gender equality, (c) promote the relative position of women, girls, and marginalized groups, including transforming underlying social structures, policies, and social norms, and (d) work to abandon the binary nature of gender (22).

**Figure 1:**
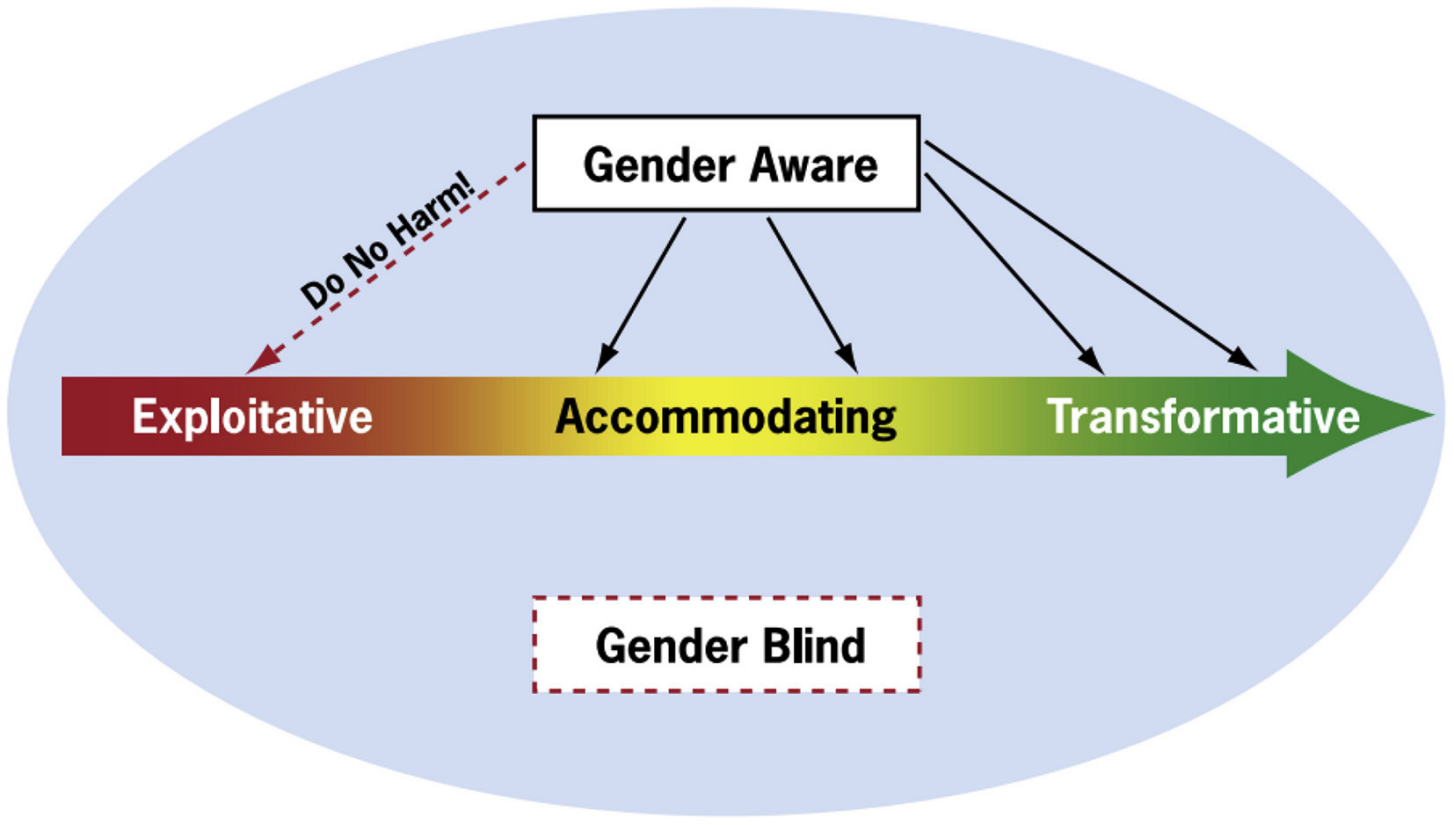
A diagram of the USAID Gender Continuum Tool designed for the ASSIST project. The tool defines each level of gender awareness on a continuum to help individuals or organizations identify not only where they fall on the continuum currently but how to reach the goal of gender transformative behaviors and policies. Source: Interagency Gender Working Group (IGWG).

Using the GECT, stakeholders were asked to identify their position along the continuum from gender blind to transformative, and post-responses on a chart. The two main categories were gender blind which ignores gender issues and dynamics affecting men and women in the LVVC; and gender aware which examines and addresses gender considerations. Gender aware institutions were further divided into three types: (i) exploitative: takes advantage of gender inequalities and stereotypes in the LVVC; (ii) accommodating: works around existing gender differences and inequalities in the LVVC; (iii) transformative: strengthens or creates systems that support gender equality (Figure 1).

### Meeting Process

Through individual and focus group discussions, mapping exercises, and presentations, participants worked together to address the research aims.

Stakeholders participated in five main activities:

1. Creation of a LVVC map in which stakeholders jointly mapped out the key actors in the LVVC, their roles, how they impact/contribute to vaccine manufacture, distribution, delivery and use, and their interactions with other stakeholders.
2. Assessing the level of gender awareness using the GECT.
3. Stakeholders' identification and classification of barriers and opportunities for women engagement along the nodes of the LVVC.
4. Outcome challenges exercises through which the stakeholders stated their current mandate, behavioral limitations, and challenges.
5. Creation of a vision status with the desired change, progress markers, and support needed.

### Ethical Approval

Informed consent was obtained prior to participating in the activities. Ethical approval for human subjects' research was obtained locally at the University of Rwanda, Office of the Director of Research and Innovation, May 2019 and in the USA, through the Tufts University Social Behavioral and Educational Research Institutional Review Board (#1907033) prior to commencement of research activities.

## Results

### The LVVC in Rwanda Is a Complex Ecosystem With Multiple Actors, Supporters, and Enablers

**Figure 2:**
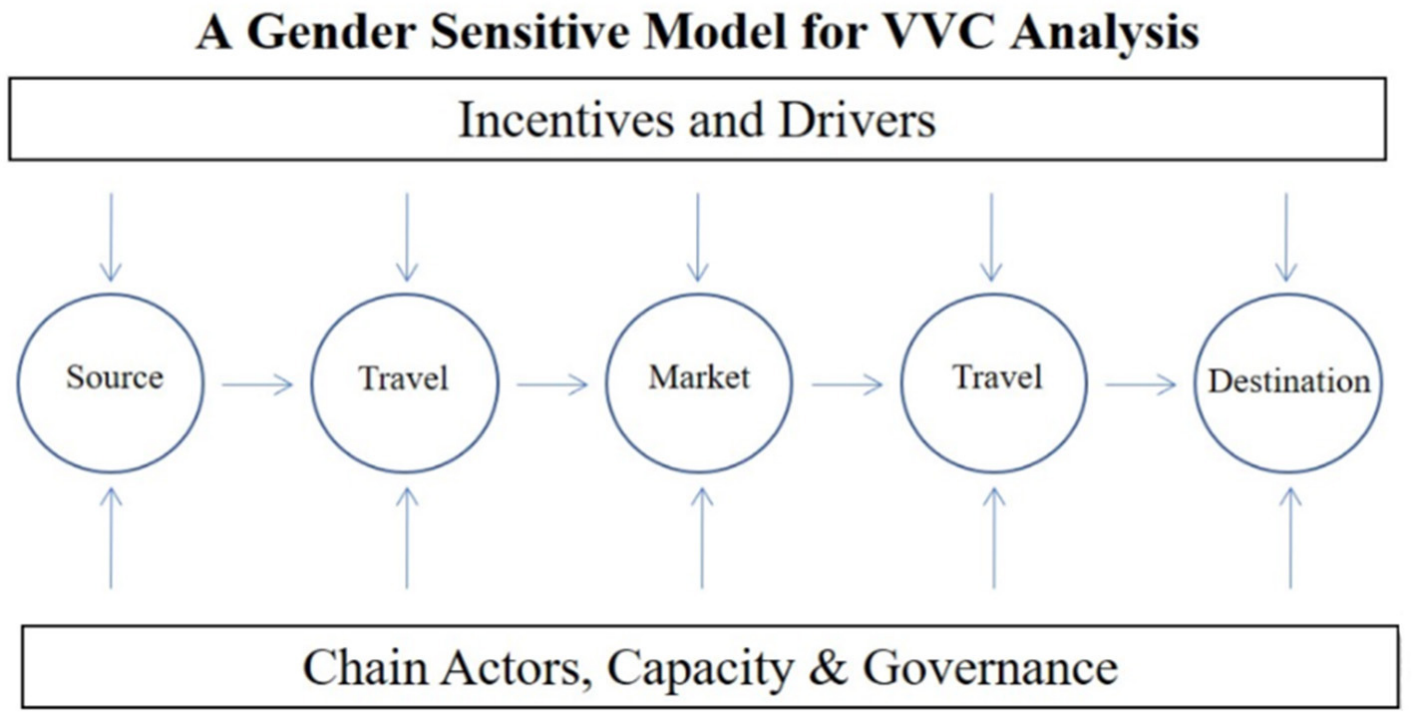
Vaccine value chain demonstrating linear movement of vaccine from manufacturer to end user.

Most LVVCs ordinarily demonstrate a linearity, with the value chain framework showing how a vaccine moves physically from the producer to the consumer, increasing in value with the nodes reflecting different actors along the chain. Figure 2 shows a standard value chain, with chain actors, whose capacities affect or influence different nodes.

Along this linear chain, there are 5 types of actors. The policy makers and regulators, manufacturers and importers of vaccines including international manufacturing companies, vaccine distributors, who range from large companies to small private companies, and include the local government, and veterinary officers who operate private agrovet businesses. Vaccine deliverers include private companies, public veterinary officers at the district and sub-county level, individual private veterinarians, and veterinary drug shop owners. Vaccine users include commercial farmers, smallholder farmers both as individuals and groups, and poultry breeding companies that supply day old chicks to farmers.

**Figure 3:**
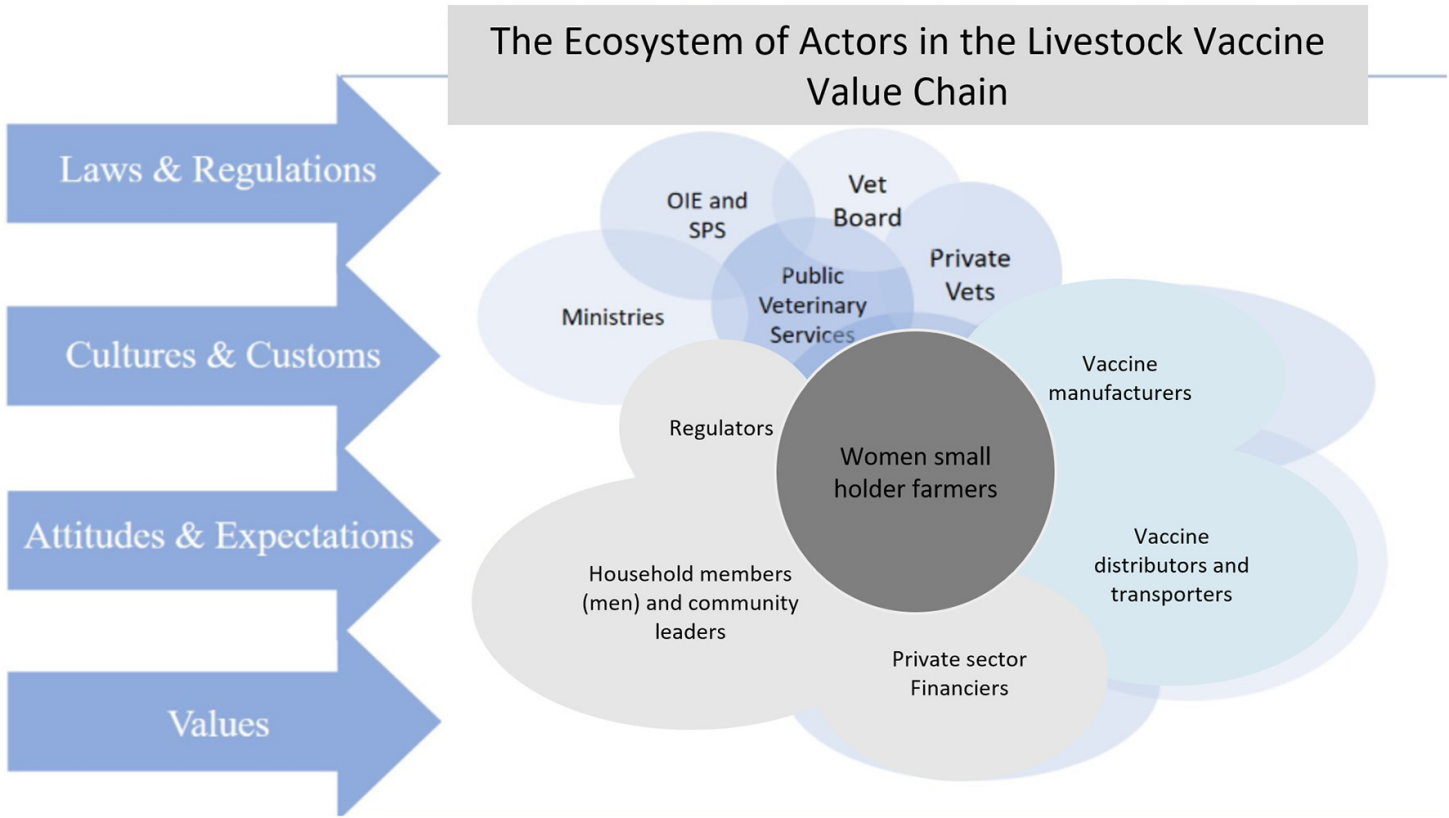
The ecosystem of actors in the Rwanda livestock vaccine value chain.

The Rwanda LVVC is not linear but exists within a complex system, an ecosystem of formal and informal actors, with different roles, incentives, drivers, capacities, and governance. In Rwanda, the main LVVC actors are the vaccine importers, distributors, suppliers, and sellers including agrovets, deliverers of vaccines; both private and public sector veterinarians, and animal health assistants, and the end users; the smallholder farmers, small scale commercial farmers, and large holder livestock farmers. However, there are many other formal and informal actors making it a complex ecosystem. These include the regulators such as the Rwanda Agricultural Board (RAB) which ensures efficient and effective quality control for manufacturing, marketing, distribution, delivery and use of livestock vaccines. Other supporting actors include the Rwanda Council of Veterinary Doctors (RCVD), farmers cooperatives (Rwanda Poultry Industry Association and IMBARAGA), livestock production groups, organizations that promote gender equality (Pro Femme Twese Hamwe), credit unions and finance institutions (Kenya Commercial Bank and Bank of Kigali), community leaders, community based organizations, local women groups, and Non-Governmental Organizations that support community development through livestock [Vétérinaires Sans Frontières (VSF) and Heifer International]. Figure 3 below shows the complexity of the Rwanda LVVC ecosystem and the interactions between different actors, resulting from the group mapping exercise.

### Stakeholder Within the Ecosystem in Rwanda Operate at Multiple Modes in the LVVC

Many of the stakeholders' actions in the LVVC ecosystem are not limited to a single node, but instead are linked to multiple nodes and provide two or more services. Many distributors also act as deliverers, selling the vaccine directly to the end users. Veterinarians own agrovet shops and act as distributors. Local farmers' cooperatives made up of different farmers groups sell livestock inputs including veterinary vaccines to their members, “*we are a local poultry association, but we also purchase and distribute feeds, vaccines and other livestock inputs for our farmers- stated a member from IMBARAGA”*. Importers sell a range of products from agricultural farm implements and products to livestock feed and pharmaceuticals. Rwanda Agribusiness Supporters and Veterinary Consulting Services LTD (RABSCO) are vaccine importers but also distribute the vaccines to local veterinarians. Individual veterinarians operating under the purview of the Rwanda Council of Veterinary Doctors (RVCD) are deliverers as they sell and administer the NCD to farmers. Academic institutions such as the EFA TVET school (an agricultural technical school), and the School of Veterinary Medicine at the University of Rwanda, sometimes implement local poultry health improvement projects, so they act as deliverers through direct vaccination, or marketing of pre-vaccinated chickens.

**Figure 4:**
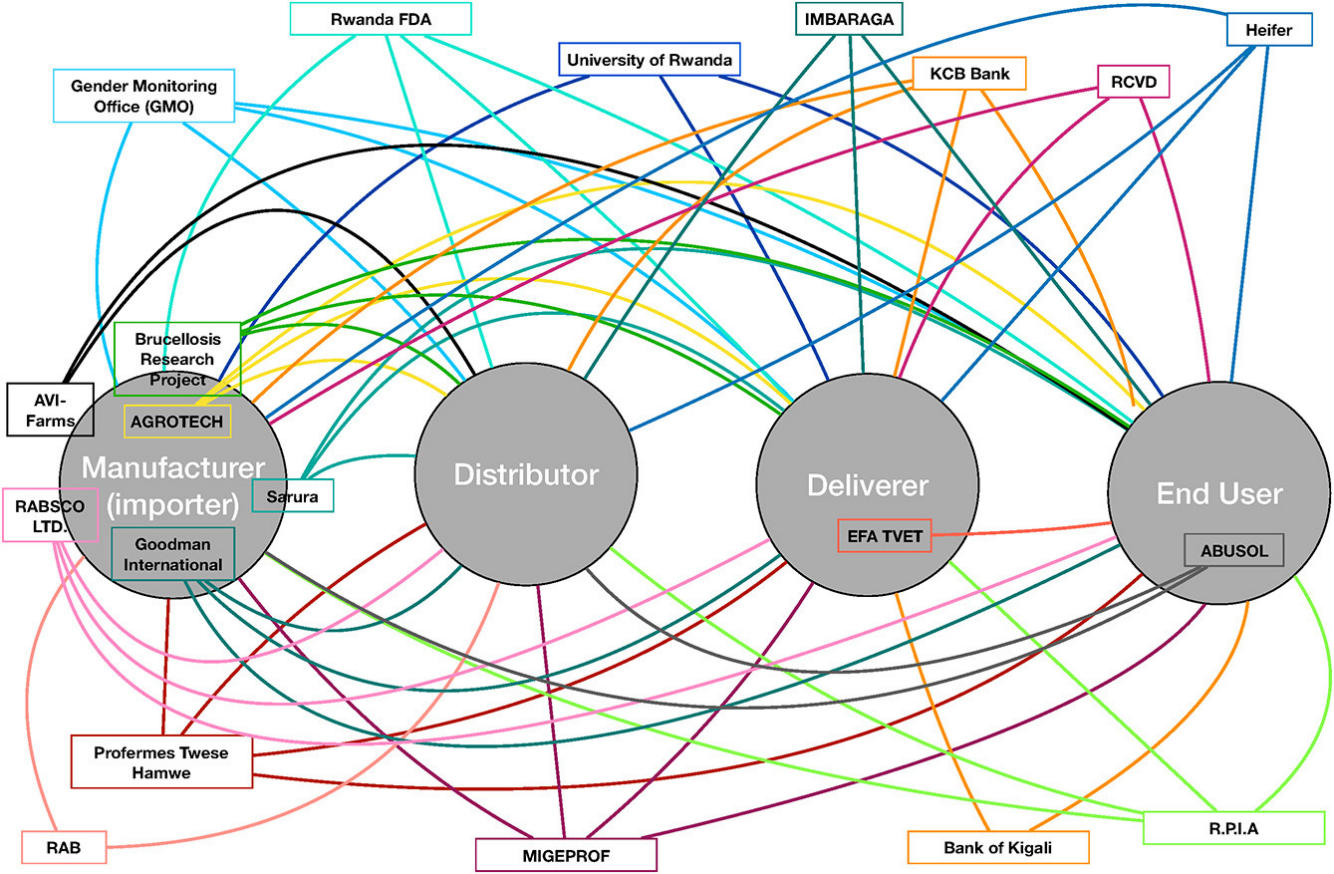
The livestock vaccine value chain ecosystem map created by stakeholders shows how different stakeholders interact at multiple nodes.

Figure 4 below shows the LVVC ecosystem map created by stakeholders and how different stakeholders interact at multiple nodes.

### The Government of Rwanda Has a Very Influential Role in Vaccine Regulation and Control

The Rwanda government members participating represented the Rwanda Agricultural Board (RAB), the Food and Drug Administration (FDA), the Rwandan Council of Veterinary Doctors (RCVD), Ministry of Gender and Family Promotion (MIGEPROF), and the Gender Monitoring Office (GMO). RAB is the main regulatory body for all agricultural inputs, while the FDA ensures quality of the manufacturing, distribution and delivery of human and veterinary drugs and vaccines. RCVD is a veterinary regulatory board that ensures that all practicing veterinarians provide quality and reliable services. The GoR and its supporting institutions develop the strategies and legislative framework that guides the manufacturing and registration of vaccines and impacts small-scale farmer's access. The state is accountable for the implementation of the relevant control measures. The dominance of the Rwandan government in the LVVC was shown not only by their sweeping influence but also in the sheer number of regulators sent from different government institutions. The tensions lie in diseases that are important at the farm or village level but not prioritized for GoR intervention. For instance, Rift Valley fever is a notifiable disease, meaning that it must be reported to the state, but all control measures including vaccination are the responsibility of the owner/s. The government provides advisory services and in certain circumstances vaccines. Any comprehensive or sustainable intervention to benefit smallholder livestock farmers, especially the women, must involve the state at national, provincial, and local levels. With a focus on boosting large scale commercialized farming, the state pays more attention to large scale farms than the smallholder farmers. Most women farmers largely fall within the smallholder farmer group and therefore do not benefit from government policies or resources.

### Most Representatives of Backyard and Small Commercial Producers of Goats and Chickens Were Female, While Those Representing Large Commercial Farms Were Male

At the end user level of the spectrum, the majority of representatives from smaller local commercial farms were female, whereas those from the larger commercial farms were male. In this set of stakeholders, men held positions of power such as director or president of farmer organizations, while women were office assistants or other supportive roles with little decision-making power. Commercial farm organizations like ABUSOL and Rwanda Poultry Industry Association, and the farmers cooperatives all had male directors while the smallholder farmer groups like Migisha Farms were represented by women at this meeting.

### Barriers and Opportunities to Women's Participation in the LVVC

#### Barriers to Women Entering the LVVC

The barriers to women's engagement with and benefits from the LVVC were classified into the following:

1. Laws and regulations.
2. Access to resources including credit, vaccines and infrastructure.
3. Cultural norms and gender stereotypes limiting women's participation in LVVC.
4. Weaknesses with vaccine distribution and training opportunities.

Table 2 presents the barriers identified at different nodes in the LVVC.

**Table 2:** Barriers and opportunities as identified by LVVC stakeholders.

**Barriers**	**Opportunities**
**Barriers and opportunities as observed by regulators**	
Limited access to skills knowledge and community outreach programs about vaccines and their use	Increase training on vaccination and usage and create easy access to information on vaccines
Government bureaucracy creates more barriers to vaccine access	Reduction in regulatory process for importation of vaccines
Vaccine packages and vial size not user friendly and not affordable for women	Work with manufacturers to create smaller dosages and package sizing for vaccines 50–100 instead of 1,000 dose vial for NCD
Inadequate cold chain	Provide solar powered refrigeration at community level for vaccine storage
Lack of investment capital/credit/collateral for women	Government focus should consider smallholder farmers and not large commercial farms only
Cultural norms and gender stereotyping limits women from engagement in animal health work	
**Barriers and opportunities as observed by importers and distributors**	
Shortage of vaccines due to restrictive government regulatory importation policies	Engage with regulatory policy makers to create conducive environment for vaccine importation and distribution
Distance to vaccine access points too long	Increase the accessibility to agrovets by encouraging more agrovets to be in the rural areas
Unreliable cold storage facilities at community level	Pursue use of solar based refrigeration or work with government to increase reliability of electricity in rural areas
Lack of necessary infrastructure for vaccine distribution	Increase access to thermostable vaccines that do not require cold storage
Lack of financial resources for smallholder farmers to purchase vaccine in bulk	Create opportunities for women smallholder to access loans and credit and other financial resources
Farmer ignorance on disease outbreaks and required vaccination	Provide training and knowledge to smallholder farmers on vaccines and diseases
Limited knowledge on vaccine use and accessibility	Provide information to farms using social media, SMS, community radio on vaccine use and where to access vaccines
**Barriers and opportunities as observed by deliverers**	
Gender promotion policies do not reach end users	Gender mainstreaming at different levels to ensure gender policies are implemented
Few females in science and veterinary schools due to admissions policies	Review laws and policies for entry into veterinary schools and make them gender responsive
Women are limited to lower-level entry and sales positions	Recognize women for their skills and competencies and create opportunities for upward mobility in the LVVC
Cultural norms, gender stereotypes and customs prevent women from holding decision making positions	Sensitize and create awareness through community engagement meetings that challenge norms and stereotypes-include husbands and community leaders
Women smallholder farmers have limited knowledge about vaccines	Build women's skills and capacities and knowledge on vaccines through training
Lack of access to training opportunities for women smallholder farmers	
**Barriers and opportunities as observed by smallholder farmers and their cooperatives**	
Traditional laws, cultures and customs limit women's roles and decision-making power	Gender awareness training for men at community level so they can provide more support for women
Very few women in decision making positions limiting advocacy for women's representation	Provide more opportunities for women to be veterinary doctors, and to work with women in the community
Cost of vaccination too high and farmers limited by small no of stock	Encourage manufacturers to reduce the packaging/vial sizes to encourage smallholder use- it is a huge market
Lack of collateral to access financial resources, loans, and credit	Creation of an entrepreneurship model for women so they can have their own supply of vaccines and access credit
Large dose formulations unaffordable for smallholder farmers	Encourage government and private sector to do a market analysis on the contribution of smallholder farmers—leading to regulations that support
Agrovets not accessible due to distance	Increase vaccine accessibility at community level by increasing no of agrovets in rural areas
Women do not have time to attend training- reproductive duties too many	Create more women friendly and accessible trainings by bringing them closer to the women
Gender stereotyping and attitudes toward women limits their participation as animal health service providers	Gender awareness training for husbands and community leaders and including them in activities so they do not feel challenged
Lack of information knowledge awareness and skills on livestock diseases and vaccines	Provide training for women in livestock disease management and vaccines
Lack of training opportunities for women	
Lack of confidence for women in handling vaccines	

##### Laws, Regulations, and Systemic Limitations

The highly centralized GoR and its broad influence on the LVVC places most activities within the purview of governmental regulatory bodies and policies. Stakeholders noted that the number of vaccines permitted to be distributed or delivered by private actors were restricted by law, and that the process of manufacturing and importing of most vaccines was limited to the government with minimal private involvement, and favored large, commercialized farms. However, the private sector was licensed to import and distribute NCD. Stakeholders noted that the government does have policies in place to promote gender mainstreaming, but they were not currently implemented, due to a lack of funding. Furthermore, academic curricula even at the veterinary school does not focus on smallholder poultry production. One veterinarian noted that “*out of the 36 credits we did in veterinary school, only one was focused on poultry. The rest are all on large animals, especially cattle. I am a poultry vet, but I had to teach myself most of what I know*”.

##### Access to Resources

Stakeholders acknowledged that the burden of poverty was difficult for both men and women, but women carried the burden of unpaid work, were busy and isolated at their homes, had fewer opportunities and less access to the resources, time, and networks needed to improve their positions, no matter how strong their desire or determination. Women wanting to start a business to distribute or deliver vaccines had less investment capital than men, and less access to loans or cold chain technology, because of a lack of collateral, and also required their husbands' permission and financial support. “*I have desired for a long time to start a business buying and selling animal vaccines because I know I would have a market, but I do not have enough collateral to get a loan from the bank*,” *one* female cooperative member said. Traditional knowledge was noted as both an opportunity and barrier among end users. Women and the very poor were more likely to rely on it because they did not have access to the money to buy modern medicine. Traditional treatment methods are not effective against viral diseases such as NCD, RVF or PPR.

##### Cultural Norms and Entrenched Stereotypes

It was noted that existing gender roles and cultural norms prevent women's active participation in the LVVC. Stakeholders noted a power differential between men and women at all points on the supply chain, which influenced positioning and decision making. They agreed that most people were not aware that women were capable of being vaccine deliverers, or veterinarians. Stakeholders observed that there was “*limited female participation, and mostly at the salesgirl level and not higher”*. Many stakeholders stated that cultural expectations for women to depend on their menfolk to make decisions resulted in low self confidence among female farmers and poor knowledge of how to access the information or support they needed. The time burden of all home and family chores prevented women from dedicating more time to improving their goat or chicken management. In some cases, livestock service providers held negative attitudes toward female farmers calling them ignorant or incapable of using modern technology. One man observed “*Norms and culture impede women from providing veterinary services, even other women expect only men to be animal health assistants and when they see a woman AHA, they do not trust them”*. The lack of professional women working as veterinarians or deliverers of information or vaccines meant there were no role models for rural girls, and very few showed interest in pursuing animal health careers. A fear of starting agricultural businesses and taking control of decisions due to risk aversion was mentioned as a key impediment to women. Since there were very few women in decision making positions this emphasized the mentality of “*give power to man and ignore woman”* which further marginalized women's needs and priorities.

##### Weaknesses With Vaccine Distribution and Training

Vaccine distributors as well as end users had little financial or technical knowledge to share with farmers in general. Poor quality of vaccines were mentioned as a common occurrence due to the limited supply of vaccines from distributors. The high number of doses in each vial, e.g., NCD vaccine, the cost, and the need for a cold chain limited smallholders' access to vaccines. It was noted that many farmers had few animals and therefore were not willing to waste their money purchasing vaccines that were packaged in volumes for large animals. One veterinarian observed “*Most of our farmers have twenty chickens, NCD vaccine is packaged in doses of 50 or 100. If they buy the whole bottle, they will be wasting at least 30 doses because they must discard the rest after opening the bottle*”. Distributors did not have the capability to maintain the cold chains all the way through to the end user, especially “the last mile” to the farm from an agrovet or deliverer/distributor, which limited access. This specifically affected women who have limited mobility. Poor access to resources was a frequently cited challenge for both women and men within the LVVC ecosystem. Women's low level of knowledge, skills, training, and resources at each node of the supply chain was noted at least 20 times as both a barrier and potential opportunity. All agreed that “*few women are trained”* at all levels, although content, cost, personnel, and purpose of training was not explored. Someone noted that “*many women do not have driving licenses”* to drive to purchase vaccines, and even then, few poor rural families have cars.

#### Opportunities Identified by Stakeholders Along the LVVC

Stakeholders agreed that a review of the laws, policies and regulations for vaccine manufacture, importation, distribution, and delivery in the country from a gender perspective was necessary to create a more supportive environment for women, as well as all smallholders, and this required political will. Technical training for end users was also a frequently mentioned solution. However, future interventions would depend on identifying successful strategies to transform social norms that prevent women from controlling resources or developing confidence in their own abilities. At the deliverer level, opportunities mentioned focused on providing skills and training in the form of capacity building for female deliverers as well raising awareness about the importance of the NCD, RVF and other livestock vaccines that are of relevance to women smallholder farmers. Table 3 presents the opportunities identified at each node.

**Table 3:** Classification of organizations along the gender equality continuum using the GECT.

**Place on gender continuum**	**Type of organization in category**	**No. of organizations**
**Gender blind**		
Organizations ignore the influence of gender and deny its effects	0	0
**Gender exploitative**		
Organizations take advantage of gender inequalities to their benefit	Pharmaceutical company, farm retailer	2
**Gender accommodating**		
Organizations identify and then accommodate or work around gender inequalities	Academia, research, retailers, farmers associations	6
**Gender transformative**		
Organizations critically examine and challenge gender inequalities attempting to make substantive change	Banks, government agencies, academia, advocacy groups, school, NGO, retailer, veterinarians' organizations	11
**Total**		**19**

### Classification of Stakeholders Along the Gender Equality Continuum

#### Discrepancies Between Perceptions and Actual Practices

Most stakeholders acting within the NCD LVVC ecosystem identified their organization's behaviors as Gender transformative due to the existence of relevant gender policies in their institutions, especially government regulators. Nearly 58% (*n* = 11) of the institutions self-identified as gender transformative. No stakeholders identified their institution as gender blind and 10% (*n* = 2) and 32% (*n* = 6) identified their organization as gender exploitative and gender accommodating, respectively. The two stakeholders identifying their organizations as gender exploitative were from pharmaceutical companies and retailers. Researchers, retailers, and representatives of farmers' organizations identified themselves as accommodating while stakeholders representing government organizations, banks, NGOs, and advocacy groups identified mostly as transformative. Table 3 shows the Classification of organizations along the gender continuum.

The reasons for self-identification as gender transformative included equal opportunity recruitment and existing gender policies. A stakeholder from a financial institution reported “*having a CEO who is a lady and who gives opportunities to other women in getting loans”* as an example of transformation. A representative of an NGO noted that men and women are “*given equal chance when it comes to staff recruitment”* and a government organization noted that they recognize “*gender equality during staff recruitment”*. Most government regulators noted “*our policies are clear about gender equality”* and that gender equality is part of their mandate (GMO and MIGEPROF). The majority of the LVVC actors identified as gender transformative when in fact their descriptions of themselves positioned them as being either gender accommodating or gender exploitative. Many of them seemed to have gender policies that were not understood, and therefore had not been translated into action. So far, they had not received any guidance on what gender equality looked like or the process of achieving it. Although progressive gender policies exist in Rwanda, stakeholders noted that lack of government explanation, definitions, implementation plan and accountability for these policies lead to non-gender transformative behaviors. There was an understanding among the stakeholders that gender policies existed especially in the government organizations. However, there was a lack of capacity to enforce or implement them, and none of the participants had any experience or exposure to gender equality training. This limited stakeholders' understanding of gender equality. This response aligned very well with information from the gender and mainstreaming youth report (12).

#### Representatives From Women's Advocacy Groups and Educational Institutions Supported More Gender Transformational Activities

Two organizations, a private TVET school and a well-established women's advocacy group were actively trying to change cultural norms/behaviors and address specific gendered issues. A school representative noted that they encouraged their “*female students to participate in all activities”* and they taught the “*role of gender [equality] in improving socioeconomic situation of families and the role of females”*, while a women's group prioritized “*advocacy around gender issues, empowering women economically, building capacity for women in leadership, fighting gender-based violence and researching gender”*.

Those stakeholders that identified as transformative felt that they were at this stage because they had “*equal numbers of women and men in positions, gender policies in place and encouraged female students”*. A participant noted that their organization had a female CEO, another noted “*clear policies about gender equality*” and that “*women and men have equal opportunities in staff recruitment and skills*”. The NGO Pro-Femmes Twese Hamwe self-identified as transformative because they were “*challenging gender norms and making substantial lasting change*”.

#### Gender Exploitative Institutions Operationalize Gender Norms to Their Benefit

In some of the organizations, it was evident that people in leadership positions were taking advantage of gender norms to exploit their female employees, according to stakeholders. One institution claimed that “*women accept lower salaries than men*” while being “*more trustworthy than men*”. They therefore preferred to hire women in lower positions and have them handle the financial sales portion of the business, while paying them less. Participants identifying their institutions as gender accommodating thought that their organizations were aware of the negative impact of gender norms but did not challenge them. They noted that their organizations may have gender policies that “*attempt to include women in decision making but women are not ready, or they do not exist*” for these roles.

### Stakeholders Perceived Challenges and Long Term Anticipated Behavioral Changes

#### Stakeholder's Baseline Status and Vision for Women's Inclusion in the LVVC

Distributors and deliverers of NCD vaccines identified changing cultural and gender norms as most essential to achieving gender transformation, while importers focused more on policy and logistics that limit access for all smallholders. According to them, female farmers' limited knowledge and skills about vaccines and low confidence resulted from few opportunities to learn. Their ideal situation was more professional women available and willing to take decision-making roles, and increasing the number of “*skilled, knowledgeable female farmers”*. The farmers' groups also noted women farmers “*lack of knowledge and problematic mindset regarding livestock vaccines”*, resulting from lack of opportunities, exposure, and female unfriendly extension services. Multiple groups characterized women farmers' baseline behavior as lacking confidence and avoiding risk.

Regulators described their institutional baseline behavior as lacking capacity for implementation and monitoring of existing gender policies. The private sector noted smallholder farmers' poor access to quality vaccines as the problematic baseline situation, in addition to profit-centric decision making.

Representatives of the veterinary, and academic sectors identified limitations as having few females as veterinary students, and researchers, and veterinarians, attributed this to cultural norms. They envisioned a “*mindset change from early childhood about gender roles”*. The researchers felt that the cultural norms and entrenched traditional stereotypes limit girls from pursuing STEM courses and resulted in fewer women in all animal sciences.

Regulators, governing agencies, and the private sector group noted good gender policies among importers and distributors, but gaps in implementation and capacity, as well as profit maximization as a guiding value. Their ideal situation could be realized from “*better enforcement of existing gender policies in government”*, and for the private sector to recognize and appreciate the market potential of small female livestock keepers.

One recurring theme was the wide recognition of both the desire to do more to ensure that women are included and participate effectively in the LVVC, but the lack of resources allocated to make this happen. Potential areas for future engagement include increasing the prioritization and therefore funding to address harmful gender and cultural norms, a system for implementation of gender policies by the government and increasing rural women's level of knowledge and skill regarding livestock vaccines. The vision statements of their end goal included increased numbers of female veterinarians and female owned businesses, greater education and skill among female farmers, reduced gender stereotypes, and improved accessibility of livestock vaccines for small scale female farmers.

##### Stakeholders Envision Change Through Advocacy and Sensitization

Stakeholders thought that empowering female livestock keepers required advocacy and sensitization. This included identification of more female role models to encourage women's involvement, advocacy for maternity leave for women and men, initiation of awareness campaigns, and training and coaching sessions for women along the NCD and RVF LVVC ecosystem. The farmer group envisioned sensitizing female livestock keepers not only around improved farming but also how to increase savings and credit. Finally, the private sector offered to “*advocate for small scale farmers by requesting that suppliers manufacture smaller dosages”* and to work with small scale farmers to access the NCD vaccine.

##### Stakeholders Suggested Solutions

One of the suggestions was to develop a veterinary vaccine “innovation platform”, because none of the institutions understood the entire vaccine value chain, and stakeholders had many false assumptions about other key actors. An innovation platform allows the stakeholders to work together as a group, creating systemic transformational change and empowerment for the women smallholder farmers through collaboration to create an enabling environment. This platform would target women's individual and collective processes to improve entrepreneurship and self-reliance, and also institutional cooperation to transform gender norms. They recognized that the current livestock vaccine system has many gaps, so coordination and understanding could lead to greater coverage and impact. For example, private sector actors could collaborate with manufacturers on solar fridge prototypes for both distributors and small-scale farmers to increase product access and quality control.

The stakeholders recommended advocacy to increase government implementation of existing gender policies, through building capacity, and establishing common standards for monitoring progress. Other suggestions ranged from maternity leave at the workplace, packaging NCD vaccine in smaller vials to make it more affordable for producers with few birds, and increased financial support and technical training for all farmers to increase demand for vaccines. They observed that advances in thermostable technology could increase accessibility and quality of vaccines according to regulators, farmers, and the private sector.

They also suggested that there was a need to develop and share policy briefs, gap assessments, political economy analysis and research/survey results from baseline data collected to influence decision making and financial resource allocation, especially from the government. Farmers and the private sector prioritized assistance to draw manufacturers' attention to small farmers as potential customers and creating farmer awareness about livestock vaccination. Multiple groups suggested training on entrepreneurship and other types of capacity building as well as on gender awareness.

## Discussion

Most livestock vaccines are available, cost-effective, and sometimes the only means to prevent infectious diseases such as RVF and NCD, however, these vaccines repeatedly do not reach, and are often not used by, women smallholder farmers (13). Vaccine accessibility, and lack of knowledge on vaccination are issues preventing increased use of livestock vaccines leading to lesser vaccine adoption in communities (13). Active involvement of women and their interaction as stakeholders in the LVVC is important to increase vaccine accessibility, demand, and adoption. Women are not well integrated into the LVVC in Rwanda according to this gendered analysis by stakeholders and their limited involvement at the end user level was evident in this analysis, yet they are perceived as critical stakeholders in the vaccine value chain, and in sustainability of livelihoods.

The control and eradication of livestock diseases is only possible with involvement of all stakeholders along the LVVC, from vaccine manufacturers and distributors, regulators deliverers and end users. According to Rathod et al. (23) global eradication of rinderpest was only possible due to the roles played by all stakeholders, including livestock owners. A holistic and sustainable model that focuses on systemic transformational change and empowerment for the women smallholder farmers is through engagement of key critical partners, analysis of the systems that are barriers across the vaccine value chain, and development of opportunities to create an enabling environment that sustains outcomes at scale.

Participants from all nodes of the supply chain are interested in strategies to improve women's position in and benefit from NCD and RVF vaccine distribution, delivery, and use. A gendered value chain approach goes beyond a straightforward supply chain map and describes the actors, institutions, regulations, and activities that bring a product from conception to its use through a gendered lens (24). The ecosystem perspective used identifies all actors within the system (both direct and supportive), then incorporates the laws and regulations, cultures and customs, attitudes, expectations, incentives, drivers, and values that surround these stakeholders and affect the flow of livestock vaccines. It emphasizes the relationships and connections among the actors that impact final delivery and use, and especially the links between private- and public-sector organizational and institutional processes. These include the systems, policies and regulations that govern vaccine availability, the different chain actors and their capacity, professional knowledge, and the gender dimensions as influenced by attitudes and culture. Engaging all the stakeholders, especially the regulatory agencies and private sector vaccine distributors and deliverers, supporting entities such as banks that deliver services to the population on a regular basis, leads to successful and sustainable community vaccine adoption. There is a need to understand the relational patterns between the various actors to ensure good governance and to address the emerging and re-emerging animal disease risks.

Drawing on qualitative data collected from key stakeholders along the LVVC in Rwanda, this study reveals an important disconnect between the perceptions of gender awareness and the practices of important players along the chain. The GECT explicitly defines gender transformation as resulting in sharing of both domestic workload and HH decision-making. Most participants seemed to understand gender equality as simply women earning income without any real changes in workload or men's behavior. GoR documents also reduce gender “equality” to adding income generation from commercialization to women's already heavy work burden. In addition, the GoR has chosen not to fund capacity building in gender within the Ministry of Agriculture from core funds, waiting instead for foreign assistance to pick it up, so it has not occurred. Despite some outspoken women, it is likely that male and also female government officials may give lip service to gender equality, but at home, do not challenge male dominance and female humility. This disconnect, which can forestall gender equality along the LVVC, is rooted not in a lack of policy but instead in translation of policies into community level practices. These findings agree with current literature regarding women's empowerment within the livestock sector in Rwanda. Lack of capacity for policy implementation has been cited by the GoR as a major roadblock to women's empowerment within the livestock sector (12), as in many other areas of the world (25). The current literature on gender transformative approaches (GTAs) in institutions notes that political will and adequate resources are essential for meaningful change. Gender transformation is at the heart of the gender continuum tool and requires a deeper understanding of what drives gender inequality beyond the top-down goals and interventions currently found in many development and government programs, especially those involving livestock. Gender transformative conversations can address the underlying gender norms that elevate men and hold women back by focusing on division of workload, leisure and decision-making, and stereotypes. However, behavior change also requires the creation of an enabling environment which supports gender responsive actions.

The “vision behaviors” of stakeholders generated by this Outcome Mapping exercise can provide direction for future interventions to improve women's participation within the LVVC. Stakeholders proposed a mindset change about gender roles in early childhood as a way to encourage more women into the profession. A number of interventions can be employed to reach these goals some of which include increasing the number of female role models within the veterinary profession and their visibility as well as increasing the exposure of young girls to science and math at a young age. Increasing the skills and knowledge of farmers by creating more farm field schools and designing a system that ensures access of women to extension services, training and credit facilities can help female farmers become more successful and empowered. Establishment of public and private partnerships to encourage cold chain access is key to vaccine accessibility. Strengthening capacity through training and shared engagement of policy makers using policy briefs, creation of stakeholder networks and financial support for intra-regional learning opportunities about vaccines and livestock for women as well as access to research regarding the market for small scale farmers in Rwanda can increase participation and benefit of women in the LVVC. These proposed interventions by stakeholders show potential to help incorporate women more along the LVVC and most importantly are synthesized and driven by those most affected along the LVVC rather than external actors.

Gender equality remains a top developmental priority as evidenced by its inclusion in the 2015 United Nations Sustainable Development goals (11). It is both an end in itself by supporting equal rights for men and women, and a means to achieve other critical goals. Women's income has a greater impact on family food security than income generated by men. The Food and Agriculture Organization (FAO) of the UN reports that women dedicate ~90% of their agricultural income to basic family needs, while men spend only 30–40% on the family (12). Targeting female subsistence farmers to both generate and control income is more effective for reducing household hunger and poverty than treating the household as a homogenous unit with equitable distribution of benefits (12). Opportunities to empower women smallholder farmers include strengthened skills and knowledge, increased economic benefits and opportunities for women to influence legal and governance structures through united voices and networks that lead to increased vaccine adoption, and eventually improved livelihoods. According to the stakeholders and given the existing challenges, the key to improving vaccine adoption among women smallholder farmers rests on setting key gender sensitive priorities and implementing and instituting the appropriate gender responsive policies and structures.

## Conclusion

Women play important roles in livestock value chains. When women own livestock, it constitutes an important component of their asset portfolio, being an asset that they can usually control, and is less contentious than land issues. However, livestock ownership in rural areas is skewed in favor of men. When women own livestock farms, men often frown upon them due to deeply rooted cultural views. Small ruminants and poultry, however, are traditionally women's animals, especially when used for food production rather than income generation. They are a good entry point for women's empowerment through improved food security and livestock productivity linked to transformation of gendered attitudes and behaviors by men and women, and supportive institutional behaviors.

Enhanced positioning and visibility of women in the LVVC, can occur through a systemic engagement of all the different stakeholders, and a recognition of the roles that women play. Women farmer involvement when determining and shaping the potential entry points is critical, given their perceived roles as being an important stakeholder in the LVVC. Viable farmer-distributor networks can be promoted and nurtured for increased vaccine accessibility; knowledge about vaccines and livestock disease prevention and control; and increased vaccine uptake.

The enhanced participation of women in livestock distribution, delivery and use can be realized by addressing identified barriers and building on the opportunities. Low vaccine uptake in particular requires robust educational/ training interventions that address the knowledge gaps. The gender capacities of LVVC actors need to be strengthened, by moving beyond the evident tokenism that is fostered by the strong yet unimplemented gender policies and embracing a more institutionalized gender transformative approach. For better results, all the intervention entry points should aim to move beyond reaching and benefitting women and foster women empowerment. This stakeholder analysis of the LVVC in Rwanda provides valuable preliminary data regarding not only the actors along the chain but also their capacities, incentives, and perceptions on strategies to increase women's inclusion in the LVVC ecosystem.

## Limitations of the Analysis

This stakeholder analysis presents valuable information about the LVVC in Rwanda, but the results must be interpreted in light of its strengths and weaknesses. It is the first attempt to research the gendered LVVC in Rwanda and is participatory at its core. The stakeholders together mapped the LVVC, identified barriers and opportunities for women, and they had an opportunity to meet, network and develop collaborations for the future.

The meeting was held in the city center of Kigali, Rwanda's capital. Although the meeting was attended by a representative from the farmer's association IMBARAGA, small-scale farmers or business owners who were unable to make the trip to Kigali were excluded. Additional stakeholder meetings will need to be held at district and village level. Other research tools such as key informant interviews and focus groups discussions will allow triangulation of the data, and an exploration of the cultural and legal assumptions brought up by stakeholders. Women's empowerment is extremely nuanced and context specific, making further probing using intersectional and qualitative tools essential.

## Data Availability Statement

The original contributions presented in the study are included in the article/supplementary material, further inquiries can be directed to the corresponding author.

## Ethics Statement

The studies involving human participants were reviewed and approved by Tufts University Social Behavioral and Educational Research Institutional Review Board (#1907033) University of Rwanda, Office of the Director of Research and Innovation. The patients/participants provided their written informed consent to participate in this study.

## Author Contributions

All authors listed have made a substantial, direct, and intellectual contribution to the work and approved it for publication.

## Funding

This research was funded by a grant from Canada's International Development Research Centre, Livestock Vaccine Innovation Fund (Grant Nos. 109061-001 and 109061-002) to Tufts University and the Africa One Health University Network (AFROHUN), under a project title SheVax+: Hearing Their Voices- Action Research to Support Women's Agency and Empowerment in Livestock Vaccine Distribution, Delivery and Use in Rwanda, Uganda, and Kenya. The Livestock Vaccine Innovation Fund was supported by the Bill and Melinda Gates Foundation (BMGF), Global Affairs Canada (GAC), and Canada's International Development Research Centre (IDRC).

## Author Disclaimer

The views expressed herein do not necessarily represent those of IDRC or its Board of Governors. LVIF website project link: https://www.idrc.ca/en/research-in-action/advancing-womensparticipation-livestock-vaccine-value-chains.

## Conflict of Interest

BM was employed by company Miller Consulting, Inc. The remaining authors declare that the research was conducted in the absence of any commercial or financial relationships that could be construed as a potential conflict of interest.

## Publisher's Note

All claims expressed in this article are solely those of the authors and do not necessarily represent those of their affiliated organizations, or those of the publisher, the editors and the reviewers. Any product that may be evaluated in this article, or claim that may be made by its manufacturer, is not guaranteed or endorsed by the publisher.
